# Commentary: Bioengineered dermal matrix reduces donor site morbidity in total phallic construction with RAFFF

**DOI:** 10.1038/s41443-024-00953-z

**Published:** 2024-07-12

**Authors:** W. G. Lee, A. N. Christopher, D. J. Ralph

**Affiliations:** 1 University College London Hospitals NHS Foundation Trust, London, UK; 2 St Peter’s Andrology, London, UK; 3 Division of Surgery and Interventional Sciences, University College London, London, UK

**Keywords:** Surgery, Quality of life

The radial artery forearm free flap (RAFFF) is the preferred flap for penile reconstruction or phalloplasty in many centres [1, 2]. Advantages of the RAFFF include the consistent anatomy, thin and pliable skin, hairless urethral segment and long vascular pedicle. These qualities allows an integrated urethral segment to be incorporated into the flap design, which is the current gold standard for urethral reconstruction [2, 3]. However, this leads to a large defect on the forearm due to the required size of the RAFFF for phalloplasty. In turn, there may be aesthetic and functional sequalae despite optimal closure that may be stigmatising to patients.

The notion of stigma is especially pertinent to transgender and gender diverse individuals. The group in Ghent, Belgium first addressed this in 2 studies reporting satisfaction of donor site reconstruction following RAFFF phalloplasty in transgender men [4, 5]. Resurfacing with split-thickness skin grafts (STSG) was compared to full-thickness skin graft (FTSG) with no difference in patient satisfaction (6.4 out of 10) [4]. Independent surgeons’ scores were also similar for both grafts though the surgeons’ scores were lower than the patients’ scores (5.7 out of 10). The second study assessed long term (median 7 years) outcomes following STSG cover [5]. Again, patients scored particular aspects of their scarring lower than dermatologists despite a satisfied or neutral score in over 75% of patients. This suggests that donor site aesthetic outcomes is a concern to patients though they may view it as an acceptable trade off in exchange for the construction of a neophallus.

Falcone et al. present a retrospective case series comparing the use of acellular dermal matrix (ADM) with STSG to their current practice of FTSG for resurfacing the forearm donor site following RAFFF phalloplasty [6]. FTSG were routinely harvested from the lower buttocks. There were no reported intraoperative complications, but operative time and partial graft loss rates were higher in the FTSG group compared to the ADM/STSG group. “Healing time” (defined as complete graft take with no further need for dressings or other treatments to the donor site) was longer in the FTSG group (30, IQR 27–34 days vs 24, IQR 20–26 days) with fewer patients reporting complete graft take following FTSG (27.8% vs 93.8%). Unfortunately, the study did not use objective assessment tools for skin quality.

Functional outcomes of donor site reconstruction following RAFFF have been assessed using more robust methods like the Patient and Observer Scar Assessment Scale v2.0 (POSAS 2.0) [7]. The POSAS 2.0 score was better following ADM/STSG though there was no difference in other questionnaires or functional assessments reported. There was also a higher satisfaction in subjective arm appearance when using ADM/STSG but not in arm functioning or the ability to work.

The concept of ADM (also called dermal regenerative templates) was born from the need to cover large full-thickness tissue defects especially where local flaps or autologous soft tissue coverage are not practical or possible. The first group offered these were acute burns patients (particularly full-thickness burns) [8]. Synthetic materials, while convenient, do not fully integrate during the healing process and are at risk of extrusion or infection as they are permanent foreign bodies. The ideal prosthesis is therefore biological or fully biodegradable allowing it to be eventually replaced or fully integrated. In addition, it should augment the body’s natural efforts, provide structural support and allow for ingrowth of tissue.

ADM are prepared by taking full-thickness section of skin from a donor. The most common donor is the human cadaver though bovine or porcine dermis is used as well. The donor tissue is processed so that cells are removed using proteolytic enzymes and detergents yielding an acellular mesh made of collagen which is non-immunogenic [8]. The ADM is placed on the donor site defect to provide a scaffold for dermal components and cells to migrate and regenerate a fully functional dermis. The matrix resorbs in the process [9].

The present study [6] used the Integra™ Single Layer dermal substitute (Integra LifeSciences Corporation, Princeton, NJ, USA) that comes without the external silicone layer. This had previously been used to successfully reconstruct large hand and forearm defects following degloving injury [10]. It is more convenient as a second operation to remove the external silicone sleeve and apply the STSG is not required.

Infections are a significant risk when using Integra™ in all types of wounds [11]. Importantly, a fatal case of toxic shock syndrome has been reported. Hence, strict infection control measures are required, including meticulous wound handling to avoid contamination during or after surgery. Antibacterial dressings and antibiotic prophylaxis may also be considered. Some centres propose aggressive protocols including topical antimicrobial dressing and frequent wound assessments. If infection is identified, daily aggressive mechanical clearance with or without systemic antibiotics are initiated. Punch biopsies are performed if invasive or deeper infection is suspected [12]. Reassuringly, this was not a significant concern in the present study.

The “elephant in the room” remains cost and unfortunately, this was not addressed by the authors with a cost-effectiveness analysis. These commercial products [13] range in price between US$5.51/cm^2^ and $ 32.93/cm^2^. The significantly higher costs of ADM by several magnitude would make it difficult for many centres to routinely use ADM with STSG for donor site reconstruction. However, the authors also did not report the rate of complications (if any) from the FTSG donor site. High levels of complications like wound dehiscence may tip the balance towards the use of ADM as was justified by Burger et al. [1]. They advocate that ADM should be considered in thin patients where a second donor site to harvest a FTSG may have higher risks of poor wound healing and excessive tension when closing.

In summary, the present study is a welcome addition to the literature on techniques for donor site reconstruction following RAFFF phalloplasty. Importantly, the benefits of ADM appear to be mostly aesthetic though there may be faster intraoperative time and postoperative wound healing when using ADM with STSG. Hence, the indications for using ADM to resurface the forearm donor site remains to be defined. Also, different ADMs have not been compared in this context so there may be cheaper alternatives that may work just as well. It is reassuring that surgical, functional and aesthetic outcomes following donor site reconstruction (with either STSG, FTSG or ADM and STSG) have improved over time with little long-term sequalae reported in the present and other recent studies.
